# Discrimination between Wild and Farmed Sea Bass by Using New Spectrometry and Spectroscopy Methods

**DOI:** 10.3390/foods11121673

**Published:** 2022-06-07

**Authors:** Giovanna Esposito, Simona Sciuto, Chiara Guglielmetti, Paolo Pastorino, Francesco Ingravalle, Giuseppe Ru, Elena Maria Bozzetta, Pier Luigi Acutis

**Affiliations:** Istituto Zooprofilattico Sperimentale del Piemonte, Liguria e Valle d’Aosta, 10154 Turin, Italy; giovanna.esposito@izsto.it (G.E.); chiara.guglielmetti@izsto.it (C.G.); paolo.pastorino@izsto.it (P.P.); francesco.ingravalle@izsto.it (F.I.); giuseppe.ru@izsto.it (G.R.); elena.bozzetta@izsto.it (E.M.B.); pierluigi.acutis@izsto.it (P.L.A.)

**Keywords:** sea bass, NIR spectroscopy, mass spectrometry, wild fish, farmed fish, discrimination

## Abstract

European sea bass (*Dicentrarchus labrax* L.) is one of the most economically important fish species in the Mediterranean Sea area. Despite strict requirements regarding indications of production method (wild/farmed), incorrect labelling of sea bass is a practice still frequently detected. The aim of this study was to evaluate the capabilities of two techniques, Near-InfraRed (NIR) spectroscopy and mass spectrometry, to discriminate sea bass according to the production method. Two categories were discriminated based on the docosahexaenoic and arachidonic fatty acid ratio by using a Direct Sample Analysis (DSA) system integrated with a time-of-flight (TOF) mass spectrometer. The cut-off value of 3.42, of fatty acid ratio, was able to discriminate between the two types of fish with sensitivity and specificity of 100%. It was possible to classify fish production by using multivariate analysis with portable NIR. The results achieved by the developed validation models suggest that this approach is able to distinguish the two product categories with high sensitivity (100%) and specificity (90%). The results obtained from this study highlight the potential application of two easy, fast, and accurate screening methods to detect fraud in commercial sea bass production.

## 1. Introduction

Fish and seafood products are considered one of the most relevant food sources of commercial interest, and in the last few years, their consumption has considerably increased, mainly because of their human health benefits [1]. Unfortunately, fishing reached unsustainable levels, exceeding, in some areas, the permitted limits. Since wild fish stocks are limited, farmed fish has been proposed as an alternative for consumers. Approximately 46% of fish come from aquaculture, and 52% of this percentage is for human consumption [2].

Expansion and improvements of the fisheries and aquaculture sector, together with greater public awareness regarding food quality, have led to a growing interest in the correct declaration of seafood, influencing world supplies and market prices. Since fish is the second category of food most vulnerable to fraud, fish authentication is important for food quality and safety assessment to control economic frauds.

Seafood labelling is an essential tool for consumer information and protection. Mislabelling information regarding the origin or production processes is increasing in frequency, and the most common falsification is the replacement of more valuable fish species with less valuable ones.

In the fishery market among the different fish species, European sea bass (*Dicentrarchus labrax*) is a fine and precious sea product, and it is one of the most frequently farmed fish of the Mediterranean countries [3]. Wild and farmed sea bass are both present in retail markets, but farmed are predominant due to the lower price. Although wild fishing is well-established in Europe, the vast bulk of sea bass production comes from aquaculture systems, where fish are bred at different densities and feed inputs.

The current worldwide interest in detecting fraudulent practices related to incorrect labelling in the production method of wild or farmed fish requires the development of reliable methods, since official methods are time and cost consuming and unsuitable for routine on-site monitoring. The ability to trace and authenticate food products is of the utmost importance for the food industry, not only for economical but also safety reasons.

Different analytical tools have been used to discriminate farmed from wild fish involving DNA analyses [4,5], chemical characteristics, fatty acid compositions [6,7], trace elements, morphology, and organoleptic characteristics [8], but only a few are related to Mass Spectrometry (MS) or Near-InfraRed (NIR) spectroscopy. Mass spectrometry studies on the differentiation between farmed and wild origins have been carried out using volatile metabolites [9] or Isotope Ratio Mass Spectrometry (IRMS) [10,11]. As previously reported in a recent study [12] by using a Direct Sampling Analysis (DSA), coupled with a time-of-flight mass spectrometer (Axion 2 TOF), it was possible to discriminate the farmed sea bream from a wild one by using a specific ratio between fatty acids. However, even if this method was very simple and rapid, currently it is necessary to develop in situ discrimination methods.

NIR spectroscopy is a widely applied technique in the fishery sector [13,14] because it can meet the need for fast and in situ analyses for food authentication. However, even though it is widely applied in food analyses [15], its application in the fish sector is usually related to discriminating between fresh and thawed sea products or fish fillet authentication. To the best of our knowledge, no studies have been developed on the sea bass production method using handheld NIR, combined with simple data processing. Over the years, miniaturized NIR has gained more importance as an alternative to classical NIR, with the advantages of minimal equipment, low cost, and easy handling, because it can be safely used even in the non-scientific community.

The aim of this study was to highlight and compare the capabilities of two methods, NIR spectroscopy and mass spectrometry, to discriminate wild and farmed sea bass without extensive sample pre-treatment.

## 2. Materials and Methods

### 2.1. Samples

A total of 90 samples of sea bass (*n* = 45 farmed and *n* = 45 wild) were bought at fishmongers and supermarkets between July 2020 and January 2021. All fish products were analysed with NIR after purchase and then stored in a refrigerator at a controlled temperature (4 °C) until MS analysis. All samples weighed within a range of 400 to 1700 g with the geographical origin of the Mediterranean area (Table 1). Three replicates were performed for each sample in NIR and MS analyses.

### 2.2. Analytical Procedure

#### 2.2.1. Extraction and Analysis of Fatty Acids

Fatty acids were extracted by using a previously reported method [12]. Sea bass muscle (5 g) was homogenized and mixed with 10 mL of n-Hexane (89% Sigma-Aldrich, St. Louis, MO, USA) and then centrifugated for 1 min at 2000× *g*. Ten microliters of each supernatant were analysed by using a mass spectrometer Axion 2 TOF, coupled with direct sample analysis (DSA) (PerkinElmer, Waltham, MA, USA) in negative mode. High resolution mass spectra were acquired in a scan range of 20–2000 (*m*/*z*) at an acquisition rate of 1 spectrum/s. The target *m*/*z* was the docosahexaenoic (22:6 ω3) [DHA-H]^−^ and arachidonic acids C20:4 ω6 [AA-H]^−^ with theoretical *m*/*z* of 327.2324 and 303.2324, respectively. Mass accuracy and peak identification were obtained by using the Axion software (PerkinElmer). The acquisition parameters were corona current 6 µA, DSA source temperature 300 °C, flight tube +10 kV, end plate 400 V, capillary exit −95 V, drying gas flow rate 1 L/min, skimmer −25 V, detector 3500 V. A standard mix solution (Agilent APCI-Santa Clara, CA, USA) infused at a rate of 10 µL/min was used to calibrate the mass peaks.

#### 2.2.2. NIR Acquisition

Spectra NIR collection and management of data were performed using the handheld SCiO device and the smartphone app (The Lab, v. 1.3.1.81) [16]. Spectra were acquired in a wavelength between 740 and 1070 nm. Each collected NIR spectrum was stored on the online Consumer Physics cloud database. Instrumental calibration was performed before the first acquisition and roughly every ten specimens following the instruction of the NIR instrument. The samples were divided into a calibration set of 78% (*n* = 70) and a validation set of 22% (*n* = 20), in order to build the models of classification and then validate them on an external data set. The spectral scans were collected in triplicate directly on the skin of each fish sample without sample pre-treatment.

### 2.3. Data Processing

#### 2.3.1. Mass Spectrometry

A total of 35 samples for each type of fish (*n* = 70) were used to create a specific correlation in terms of a fatty acid ratio value capable of discriminating between wild and farmed sea bass. The mass spectra scans were collected in triplicate for each fish sample. The intensity of *m*/*z* spectra was recorded and used to determine the ratio of fatty acids. Data analysis was performed using STATA 15.1. statistical software. A non-parametric linear (local) regression model was applied [17] because an assumption of normality of distribution was not fulfilled. A non-parametric receiver operating characteristics (ROC) curve [18] was estimated to define a fatty acid ratio value that could effectively discriminate between farmed and wild sea bass. In order to construct the ROC curve, farmed and wild sea bass were considered as negative and positive samples, respectively. Method performances were evaluated by determining repeatability expressed as relative standard deviation (RSD%), sensitivity, specificity, and accuracy.

#### 2.3.2. NIR Spectroscopy

Principal component analysis (PCA) was performed, without applying data pre-treatments in which the samples were not effectively discriminated.

All the collected spectra were processed according to the four pre-defined algorithms (“Processed”, “Normalize”, “Processed & Normalize”, and “(log)R & Normalize”) present in the SCiO app [16]. Data pre-treatments were used to reduce the physical variability between samples due to scatter and to adjust baseline shifts and drift. Below is the sequence of pre-treatments used in each algorithm:

- Processed: log, average scan, 1st derivative and 2nd order polynomial, select range (nm), subtraction average.
- Normalize: average scan, select range (nm), standard normal variate (SNV).
- Processed & Normalize: log, average scan, 1st derivative and 2nd order polynomial, select range (nm), SNV.
- (log)R & Normalize: log, average scan, select range (nm), 2nd derivative and 2nd order polynomial, SNV.

The results obtained were expressed in terms of generated calibration models and were compared for coefficient of variation (f1) and matrix of confusion. The models were subsequently used to test the validation set and compared in terms of sensitivity, specificity, and accuracy. Farmed and wild sea bass were considered negative and positive samples, respectively.

## 3. Results and Discussion

### 3.1. Fatty Acids Ratio

**Figure 1:**
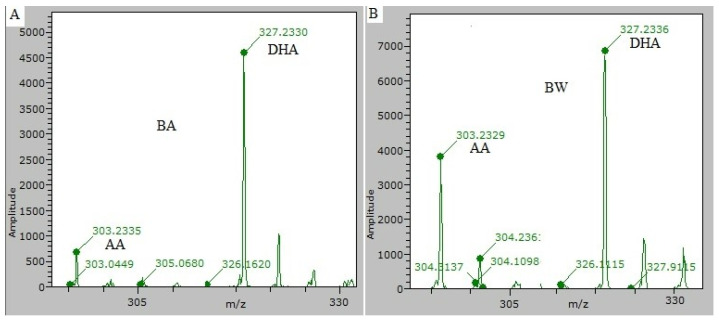
[DHA-H]^−^ and the [AA-H]^−^ mass spectrum obtained from farmed (BA) (**A**) and wild (BW) (**B**) sea bass, respectively. Errors in mass identification were less than 2 ppm and scores were higher than 0.71.

The adducts of fatty acids [DHA-H]^−^ and [AA-H]^−^ selected as markers to discriminate wild (BW) from farmed (BA) sea bass were correctly identified in the sample extracts (Figure 1) with a small error in mass identification (ppm) and a good probability (score) to correctly identify these molecules.

In detail for [DHA-H]^−^, an error between 0.26 and 2.4 ppm and a score between 0.686 and 0.949 were obtained, while for [AA-H]^−^, an error between 0.121 and 2.01 ppm and a score between 0.702 and 0.852 were obtained.

**Figure 2:**
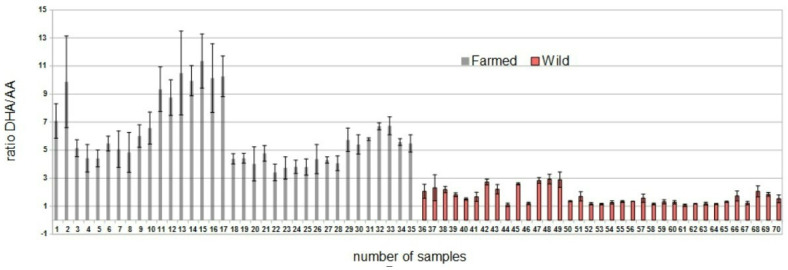
Fatty acids ratio values in sea bass samples analysed under the same conditions. Each value is an average of triplicate analyses (with SD).

In order to compensate for species variability and discriminate between wild and farmed fish, we compared the two classes of fish based on the ratio between the intensity of the [DHA-H]^−^ and the [AA-H]^−^ mass peak (*m*/*z*). In detail, the ratio between the two adducts for the 35 BW samples was within a range of mean values between 1.07 (SD 0.08) and 2.93 (SD 0.34), while the ratio between the two adducts for the 35 BA samples was within a range of 3.42 (SD 0.59) and 11.06 (SD 1.93) (Figure 2).

A similar difference between this ratio was obtained in a paper where the total fatty acids profile was studied in sea bass [19]: the DHA/AA ratio obtained from the values recorded was around 4.67 and 12.67 for wild and sea bass, respectively. The high variability in the ratio of fatty acids in farmed fish can be a consequence of the type of feeding available in the aquaculture market.

### 3.2. Statistical Analysis

#### 3.2.1. Mass Spectrometry

The results obtained from the mass spectrometry descriptive statistical analysis suggest the presence of differences between BW and BA samples; the distribution of the DHA/AA shows the mean ratio for BA (6.16; SD 2.3) higher than the BW (1.69; SD 0.58).

The non-parametric model estimated a statistically significant (*p*-value < 0.001) average effect of farmed bass about 4.36 units of DHA/AA. The goodness of fit of the model is equal to R^2^ = 0.60.

The differences in DHA/AA between the two groups could be probably explained by the differences in feeding. In the last decade, the feeding industry has promoted the use of ingredients for aquaculture feeding that can improve the levels of ω3 fatty acids and in particular DHA [20]. However, some studies reported the importance of supplying fish diets not only with ω3-3 PUFA but also with ω6-PUFA to improve growth performance and immune system function [21]. Consequently, the low ratio of DHA/AA could derive from the major assumption of feed that contains AA in BW compared to BA, as well as the large size of BW with respect to the BA.

Regarding the determination of the cut-off, since the maximum DHA/AA value of the BW (i.e., 2.93) is lower than the minimum DHA/AA value of the other type of subjects (i.e., 3.42), any value between these two is able to perfectly discriminate between the two types (i.e., with sensitivity = 100% and specificity = 100%). With regard to the ROC curve, the estimated area under the curve (AUC) was 100%.

As the previous study confirms [12], using DSA-APCI-TOF-MS, it is possible to differentiate farmed from wild fish using the fatty acid ratio. The repeatability RSD% was less than 15% for BA and BW, confirming that this marker is very useful in this field of application.

#### 3.2.2. NIR Spectroscopy

The first explorative analysis to evaluate fish classification was the principal component analysis (PCA). Without applying data pre-treatments, samples were not effectively discriminated. Subsequently, the four algorithms for classification presented in the app were used to create calibration models. Data from SCiO were generated in the diffuse reflectance mode, which can be affected by light scattering spectroscopy artefacts that are not of interest for the characterization of the samples under study, but can interfere with the right classification. Therefore, pre-treatments of data are necessary to apply in order to reduce or eliminate these inconveniences. In this analysis, the following were applied: the first derivative to adjust the baseline, the second derivative to adjust both baseline and linear trend, SNV for scatter correction, and log to force the data to obey the Lambert–Beers law. The best results in classification analysis were obtained by reducing the spectral range between 980 and 900 nm.

As shown in Table 2, the confusion matrices obtained by using the four pre-defined algorithms showed minor disparities in fish production classification. Although the “processed” algorithm showed the most efficient result in term of classification, all models were tested with the validation set samples.

The results achieved by the validation test suggest that all the models were able to distinguish the two product categories (Table 3) within a range of sensitivity, specificity, and accuracy of 100%, 70–90%, and 90–95%, respectively.

According to the models obtained, 3 samples were not correctly classified: with the processed algorithm one sample BA was classified as BW, and two samples were classified as undefined; with Processed & Normalize, two BA were classified as BW, and one was classified as undefined; with Normalize and (log)R & Normalize algorithm, one BA sample was classified as undefined.

Considering practical application, by using this simple and rapid NIR device, it was possible to classify fish products according to the type of production with good performance, without extensive and elaborate chemometric analysis. In addition, by using a portable NIR, it is possible to detect frauds regarding wild/farmed seafood even by inexperienced personnel and directly in situ.

## 4. Conclusions

The results obtained from this work demonstrate the feasibility of two simple and rapid methods to discriminate the type of production of sea bass using NIR spectroscopy and mass spectrometry.

Both methods allowed for the discrimination between farmed and wild sea bass with minimal or no chemical treatments and with sensitivity, specificity, and accuracy higher than 90%.

The main advantages of the proposed study over the traditional methodologies of food analysis are, for mass spectrometry, the possibility of analysing the samples directly after a simple extraction procedure, without the use of HPLC separation and without the use of the internal standard to quantify the fatty acid marker. Instead, the rapidity of acquisition, the low cost of portable NIR, and the ease in creating models with NIR spectroscopy suggest the advantages of using this approach in routine operations to implement fraud control, in terms of fish production, directly in situ.

## Figures and Tables

**Table 1:** Geographical origin, weight, and sample number of farmed and wild sea bass.

	FARMED SEA BASS				WILD SEA BASS	
**Geographical origin**	Croatia	Greece	Oriental Mediterranean	Italy	Oriental Mediterranean	Italy
**Weight (g)**	400–600	400	650	500–750	800–200	650–1700
**Number of samples**	15	8	15	7	15	30

**Table 2:** Classification results: farmed/wild sea bass. All values are expressed in percentage.

Model	Performance f1	Predicted BA (%)	Predicted BW (%)
Processed	0.88	86	91
Normalize	0.84	86	81
Processed & Normalize	0.83	84	86
(log)R & Normalize	0.80	76	85

**Table 3:** Classification rate in prediction set with the four models for farmed/wild sea bass.

Model	Performance f1	Class	Classification Rate per Class %	Overall Classification Rate %
Processed	0.89	BA	70 (3/10)	85 (17/20)
		BW	100 (10/10)	
Normalize	0.97	BA	90 (1/10)	95 (19/20)
		BW	100 (10/10)	
Processed & Normalize	0.87	BA	70 (3/10)	85 (17/20)
		BW	100 (10/10)	
(log)R & Normalize	0.97	BA	90 (1/10)	95 (19/20)
		BW	100 (10/10)	

## Data Availability

Data available on request.
